# Obésité abdominale et autres biomarqueurs de risque cardiométabolique: influence du niveau socioéconomique et du mode de vie dans deux populations noires apparentées, Cotonou (Bénin) et Port-au-Prince (Haïti)

**DOI:** 10.11604/pamj.2016.24.306.8530

**Published:** 2016-08-10

**Authors:** Asma EL Mabchour, Hélène Delisle, Colette Vilgrain, Phillipe Larco, Roger Sodjinou

**Affiliations:** 1 TRANSNUT, Centre Collaborateur de l’OMS sur la Transition Nutritionnelle, Département de nutrition, Faculté de médecine, Université de Montréal, Québec, Canada; 2 Fondation Haïtienne de Diabète et des Maladies Cardiovasculaires (FHADIMAC), Port-au-Prince, Haïti; 3 Organisation Ouest Africaine de la Santé (OOAS), Bobo-Dioulasso, Burkina Faso

**Keywords:** Abdominal obesity, cardiometabolic risk, waist circumference cut-off values, Abdominal obesity, cardiometabolic risk, waistline-threshold values, Africa, Caribbean, nutrition transition

## Abstract

**Introduction:**

L’augmentation du risque cardio-métabolique (RCM) dans les pays à faible et à moyen revenu résulte pour large part d’une transition nutritionnelle rapide. Cette étude est réalisée dans deux groupes de population apparentés mais vivant dans des environnements différents. Elle vise à cerner la relation entre le mode de vie et les biomarqueurs de RCM, ainsi qu’entre l’obésité abdominale (OA) et les autres biomarqueurs.

**Méthodes:**

L’étude porte sur 200 Béninois de Cotonou et 252 Haïtiens de Port-au-Prince (PAP) âgés de 25 à 60 ans et apparemment en bonne santé. L’OA est définie spécifiquement par un tour de taille (TT) ≥ 88cm (hommes) et ≥ 95cm (femmes). Les autres biomarqueurs les plus fréquents sont considérés: un ratio cholestérol total/HDL-cholestérol élevé, une tension artérielle élevée et la résistance à l’insuline d’après HOMA (Homeostasis Model Assessement). Le niveau socio-économique (NSE), l’alimentation, la consommation d’alcool et de tabac sont documentés par questionnaire. Deux schémas alimentaires ressortent de l’analyse typologique, l’un traditionnel et l’autre « transitionnel », avec une fréquence accrue d’aliments occidentaux.

**Résultats:**

Le NSE, la consommation d’alcool et le tabagisme sont associés au RCM, mais non le schéma alimentaire. L’OA est associée aux autres biomarqueurs de RCM, sans effet marqué du NSE et des variables du mode de vie.

**Conclusion:**

Les valeurs-seuilsspécifiques de TT se confirment. Aussi, le NSE et le mode de vie influencent le RCM mais non la relation entre l’OA et les autres biomarqueurs de RCM.

## Abstract (en)

**Introduction:**

Increased cardio metabolic risk (CMR) in low- and middle-income countries is largely due to rapid nutrition transition. We conducted a study of two African-origin populations groups living, however, in widely different settings. It aimed to assess the relationship between lifestyle and CMR biomarkers as well as between abdominal obesity (AO) and other biomarkers.

**Methods:**

The study included 200 Benineses from Cotonou and 252 Haitians from Port-with-Prince (PAP) aged between 25 to 60 years and apparently in good health. AO was specifically defined as waist circumference ≥ 88cm (men) and ≥ 95 cm (women). Other most common biomarkers were: high total cholesterol/HDL cholesterol ratio, high blood pressure and insulin resistance by HOMA (Homeostasis Model Assessement). Socioeconomic status, diet, alcohol and tobacco were documented by questionnaire. Two dietary patterns emerged from cluster analysis, one traditional and the other "transitional" with increasing frequency of western foods.

**Results:**

Socioeconomic status, consumption of alcohol and nicotinism were associated with CMR, but not the food diagram. AO was associated with other CMR markers, with no marked effect of socioeconomic status and lifestyle variables.

**Conclusion:**

Specific TT threshold values are confirmed as socioeconomic status and lifestyle have an impact on CMR, but not the relationship between AO and other CMR biomarkers.

## Introduction

Les changements dans le système alimentaire mondial ont précipité les pays en développement dans une transition nutritionnelle caractérisée par une alimentation qui s’occidentalise et un mode de vie de plus en plus sédentaire, surtout en ville; à cela s’ajoute l’influence du niveau socio-économique (NSE), du contexte culturel et des facteurs génétiques individuels, facteurs qui contribuent tous à moduler la prévalence de l’obésité et du risque cardiométabolique (RCM)dans les populations et chez les individus [1–3]. Plusieurs études confirment la relation entre l’obésité abdominale (OA) et les autres biomarqueurs de RCM, notamment l’insulino-résistance, la dyslipidémie et l’hypertension [4]. Cependant, rares sont les travaux sur cette association dans des populations subsahariennes et apparentées [5, 6]. Plus récemment, la pertinence de définir des valeurs-seuils de tour de taille (TT) spécifiques aux populations africaines pour prédire les aberrations cardiométaboliques a été évoquée et quelques études en ce sens confirmaient que les valeurs-seuils établies chez les Caucasiens n’étaient pas appropriées pour les Africains [7–10]. Toutefois, on ne relève pas d’études explorant l’influence du (NSE) et du mode de vie d’une part sur l’occurrence de l’OAdéfinie par des valeurs-seuils spécifiques et d’autre part, surla relation entre l’OA et les autres biomarqueurs de RCM fortement prévalent dans des populations africaines et apparentées. C’est l’objet de notre étude à Cotonou (Bénin) et Port-au-Prince (Haïti). Après avoir défini des valeurs-seuils spécifiques de TT pour prédire les biomarqueurs de RCM les plus fortement répandus dans ces deux groupes de population génétiquement proches mais vivant dans des contextes très différents [10], nous nous penchons ici sur les interrelations entre le mode de vie et le NSE d’une part, l’OA et les autres biomarqueurs de RCM d’autre part.

## Méthodes

Il s’agit d’une étude transversale chez 200 adultes (50 % d’hommes) vivant à Cotonou, la capitale économique du Bénin, et chez 252 adultes (541 % d’hommes) de Port-au-Prince, la capitale d’Haïti. Les sujets, âgés de 25 à 60 ans, sont apparemment sains, sont issus de parents et de grands-parents noirs et résident en ville depuis au moins six mois afin d’exclure l’influence d’une récente migration urbaine, laquelle est démontrée [**3**]. Les données sont collectées à Cotonou en 2005 et à PAP seulement en 2008 en raison de problèmes de sécurité. L’échantillonnage aléatoire en grappeset la population de l’étude dans les deux villes sont décrits ailleurs [10, 11].

### Variables biologiques et biomarqueurs de risque cardiométabolique

Les techniques de mesure de la tension artérielle et des paramètres anthropométriques (poids, taille et TT) ont été harmonisées entre les deux villes. Quant-aux analyses biochimiques, elles ont été réalisées au même laboratoire (Nancy- France)au moyen des mêmes méthodes afin de permettre des comparaisons. Les biomarqueurs de RCM considérés dans la présenteétude sont ceux présentant uneforte prévalence dans ces groupes de population [12]: l’OA, un indice élevé d’athérogénicité, l’insulino-résistance et une tension artérielle élevée. L’OA est définie par un TT = 95cm chez les femmes et = 88cm chez les hommes, seuils qui prédisent le mieux, pour l’ensemble des sujets de Cotonou et de PAP, au moins deux des trois biomarqueurs les plus fortement prévalents avec une spécificité de 80 % [10]. Le TT est mesuréà mi-chemin entre la dernière côte et la crête iliaque, à 0,1cm près [11, 13]. La moyenne de deux mesures de TT est utilisée dans les analyses. **Un ratio d’athérogénicité élevé** correspond à un rapport des concentrations sériques du cholestérol total et des lipoprotéines à haute densité (CT/HDL-C)>5 chez les hommes et >4 chez les femmes [14]. **L’insulino-résistance** est définie par le75^ième^ percentile de HOMA (Homeostasis Model Assessment), pour l’ensemble de la population (3,9), calculé selon la formule: (glycémie à jeun x* insulinémie à jeun)/22,5 [15]. **Une tension artérielle élevée** répond aux critères de la Fédération Internationale de Diabète (FID), soit une tension systolique (TAS) = 130mm Hg ou une tension diastolique (TAD) = 85mm Hg [16]. La technique standard est utiliséeet la moyennedes deux mesures sert dans les analyses.

### Niveau socio-économique, habitudes alimentaires et mode de vie

Dans chaque ville, un questionnaire prétesté auprès de 10 sujets ne participant pas à l’étude et administré par des interviewers formés sert à déterminer le NSE (proxy du revenu et niveau d’éducation), la fréquence de consommation d’aliments sélectionnés, ainsi que la consommation d’alcool et de tabac. Les questions sur la consommation d’alcool et de tabac sont tirées du questionnaire STEPS de l’OMS pour les enquêtes sur les facteurs de risque des maladies non transmissibles [17]. Un score servant de proxy du revenu est calculé par analyse en composantes principales (ACP) [18], à partir des biens durables possédés par les ménages, des services dont ils bénéficient et des caractéristiques du logement, comme il est difficile de cerner le revenu dans les populations de pays en développement [19]. Les éléments considérés sont adaptés des enquêtes démographiques et de santé (EDS) menées au Bénin [20] et en Haïti [21]. Douze éléments sont finalement pris en compte suite aux analyses descriptives, dont neuf sontcommuns aux deux villes. Trois élémentsdiffèrentselon le contexte : à Cotonou, la possession d’une motocyclette ou d’un téléphone fixe et le type de matériaux des murs; à PAP, la possession d’un vélo, la vie à l’étranger d’un proche parent et un séjour à l’étranger d’un membre du ménage au cours des cinq dernières années, car une bonne partie des revenus provient de transferts d’argent par des proches résidant hors d’Haïti [21]. Tous les éléments sont dichotomiques (0/1 pour non/oui). Le score de revenuainsi obtenuest subdivisé en trois terciles dans chaque ville (faible, moyen et élevé). Le niveau d’éducation correspondau plus haut niveau de scolarité atteint, en trois catégories : 0, pour aucune instruction formelle, 1, pour le niveau primaire et 2 à partir du secondaire. Lesschémas ou modèles alimentaires sont définis à partir de la fréquence de consommation au cours de la semaine écoulée de 33 aliments à PAP et de 26 aliments à Cotonou. Les aliments avaient d’abordété regroupés par l’équipe de chercheurs en aliments occidentaux comme les boissons gazeuses, les bonbons, le chocolat, etc., les aliments typiquement urbains et les aliments traditionnels dans les deux contextes (Tableau 1). Les données sontd’abord transformées en cotes-Z et l’analyse typologique (cluster analysis) utilisant la méthode de classification par nuées dynamiques (k-means) classe ensuite les individus dans des catégories mutuellement exclusives [22]. Deux à huit classes sont choisies consécutivement pour ces analyses itératives. La consommation d’alcoolest évaluée par un score composite comprenant la quantité totale d’alcool ingérée dans la semaine en grammes/jour et la dose maximale consommée en une prise, tel que suggéré par l’OMS [23]. Le score varie entre 0 pour aucune consommation et 4 pour une consommation élevée avec un alpha de Cronbach standardisé de 0,95 à PAP et de 0,93 à Cotonou. La quantité quotidienne moyenne d’alcool pur tient compte du degré alcoolique, du volume des différentes boissons commerciales ou artisanalesconsommées en plus de la densité de l’éthanol (0,79 g/ml) [23]. Pour juger d’une consommation sporadique élevée, la dose maximale en une seule occasion (bingedrinking) est = 40 g d’alcool pur chez les femmes et = 60 g chez les hommes [24, 25]. Les sujets ont été classés en quatre catégories de consommation d’alcool: 0, nulle; 1, faible à modérée, ou sporadique à dose non excessive (= 15g/jour pour les femmes et = 30g/jour); 2, faible à modérée mais sporadique élevée; et 3, élevée (> 15 g/jour pour les femmes ou > 30 g/jour pour les hommes, outre une consommation sporadique élevée). Les sujets sont classés en trois groupes pour le tabagisme: 0, les non-fumeurs; 1, les anciens fumeurs (ayant cessé de fumer depuis au moins six mois); et 2, les fumeurs.

**Tableau 1:** Fréquence de consommation des aliments en fonction des modèles alimentaires; moyenne ± écart-type

	Port-au-Prince			Cotonou		
	Traditionnel (n=158)	Transitionnel (n=86)	p	Traditionnel (n=139)	Transitionnel (n=48)	p
***Aliments occidentaux***						
Boissons gazeuses	0,87±1,50	3,19±3,26	<0,001	0,50±0,88	1,96±2,50	<0,001
Jus artificiel	0,22±0,60	0,76±1,46	0,002			
Boisson énergisante	0,20±0,64	0,64±1,39	0,008			
Bonbons	0,45±0,95	1,54±2,03	<0,001	0,55±1,02	1,10±1,78	0,047
Chocolat	0,04±0,28	0,39±0,87	0,001	0,01±0,12	0,08±0,28	0,103
Frites	0,04±0,22	0,20±0,59	0,018	0,06±0,25	0,21±0,46	0,044
Hot dog	0,18±0,70	0,59±1,24	0,006	0,02±0,15	0,10±0,37	0,131
Pâtisseries	0,04±0,24	0,24±0,60	0,005	0,38±0,85	0,58±0,94	0,168
Glaces	0,12±0,50	0,29±0,63	0,032	0,11±0,39	0,10±0,47	0,957
Hamburger				0,02±0,15	0,33±1,00	0,036
***Aliments urbains***						
Pâtés de viande	0,04±0,24	0,27±0,54	0,001			
Pâtés de poulet	0,05±0,22	0,32±0,59	<0,001			
Mets frits de rue	0,29±0,73	1,43±2,15	<0,001			
Biscuits	0,11±0,63	0,83±1,94	0,002	0,43±0,79	1,38±2,08	0,003
Charcuteries				0,21±0,60	0,63±1,33	0,041
Conserves de poissons				0,13±0,38	0,83±1,40	0,001
Fromage				0,12±0,44	1,35±2,34	0,001
***Aliments traditionnels***						
Marinade^1^	0,15±0,50	0,85±1,83	0,001			
Riz sauce pois^2^	1,91±1,44	3,20±1,92	<0,001			
Riz collé^3^	2,54±1,93	3,65±3,05	0,003			
Légume-viande	1,47±1,38	1,88±1,71	0,044			
Viande et sauce^4^	1,44±1,32	2,76±2,08	<0,001			
Plantain frit	0,36±0,80	0,94±1,59	0,002			
Pain	3,30±3,40	5,98±3,99	<0,001			
Café	2,17±2,60	3,79±2,92	<0,001			
Bouillon^5^	0,42±0,67	0,76±0,95	0,005			
Soupe^6^	0,07±0,30	0,19±0,42	0,022			
Ablo/Com^7^				0,42±1,00	0,15±0,36	0,007
Agbéli^8^				0,05±0,25	0,58±0,96	<0,001
Eba^9^				0,24±0,60	0,54±0,92	0,041
Téloubo^10^				0,17±0,44	1,17±1,52	<0,001
Agou^11^				0,13±0,40	0,58±1,01	0,004

Seuls les aliments dont les fréquences de consommation sont significativement différentes entre les deux modèles sont présentés; Les valeurs soulignées sont significativement plus élevées

1 beignets de farine de blé

2 riz blanc+saucede purée de haricots

3 riz cuit avec haricots rouges (riz national)

4 sauce généralement à base d’oignions et de tomates

5 viande, légumes, igname, etc.

6 giraumon, carotte, etc.

7 gâteau de maïs fermenté et sauce (légumes, viande/poisson, huile d’arachide/palme)

8 pâte de manioc fermentée et sauce

9 farine de manioc et sauce

10 pâte de cossette d’igname avec sauce

11 igname pilé et sauce tomate

Les analyses statistiques sont effectuées à l’aide du logiciel SPSS version 22 (Armonk, NY: IBM Corporation). Les variables catégorielles sont analysées par le test de Khi 2 et les variables continues, par le test de t de Student. Les associations entre les variables de NSE ou demode de vieet lesbiomarqueurs de RCM, comme entre l’OA et les autres biomarqueurs (en tenant compte du NSE et du mode de vie), sont analyséespar régression logistique multiple. Les courbes ROC «Receiver Operating Characteristics» pour vérifier la valeur prédictive de l’OA vis-à-vis des autres biomarqueurs de RCM, en tenant compte ou non du NSE et du mode de vieet en contrôlant pour l’âge et le sexe, sont générés et les aires sous la courbe (ASC) comparées (à l’aide du logiciel Medcalc version 15) dans chaque ville. Plus l’ASC tend vers 1, meilleur est le pouvoir prédictif du test [26]. Le niveau de signification statistique est fixé à p < 0,05.

### Considérations éthiques

Les études primaires ont été approuvées par le comité d’éthique et de recherche de la Faculté de Médecine de l’Université de Montréal, par le Ministère de la Santé Publique et de la Population (MSPP) de la République d’Haïti et par le Ministère de la Santé Publique de la République du Bénin. Tous les participants ont signé un formulaire de consentement éclairé. Les sujets chez qui une hypertension artérielle ou une dysglycémie sont détectées sont référés à un médecin spécialiste pour diagnostic au frais du projet. Les résultats obtenus ont été partagés avec les organismes partenaires des deux pays.

## Résultats

**Figure 1:**
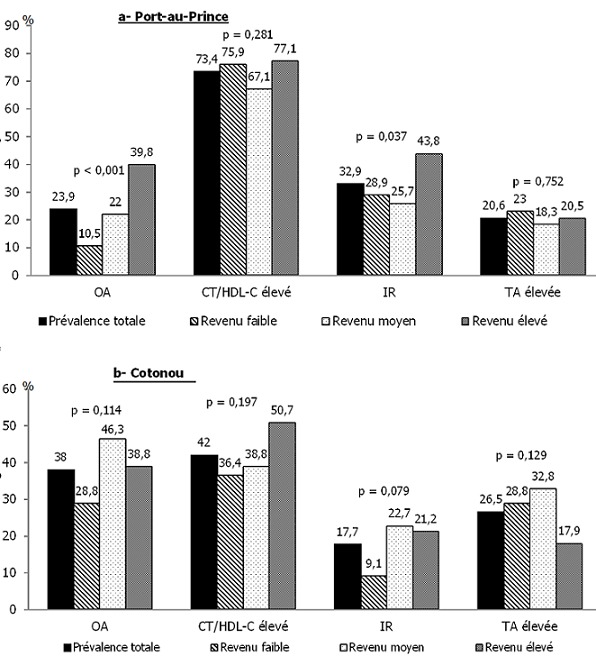
Prévalence des biomarqueurs de risque cardiométabolique en fonction du revenu

Les données socio-économiques, comportementales et biologiques sont présentées au Tableau 2. Le niveau d’éducation est plus élevé à PAP qu’à Cotonou. Chez les femmes, la consommation d’alcool est plus élevée à Cotonou mais le tabagisme est plus répandu à PAP. Chez les hommes, il n’y a pas de différences significatives entre villes pour la consommation d’alcool ou de tabac. Les données anthropométriques, biochimiques et biologiques montrentdes différences significatives entre villes tant chez les hommes que les femmes, excepté pour le TT, qui ne varie pas de manière significativechez les femmes. Sur l’ensemble des sujets et en comparant le profil cardiométabolique à PAP et Cotonou, l’OA d’après les seuils de TT définis pour cette population (= 88cm chez les hommes; 95cm chez les femmes) est de 38 % à Cotonou et de 23,9 % à PAP (p = 0,001); les fréquences totales des autres biomarqueurs de risque au niveau des villes sont aussi illustrées à la Figure 1.

**Tableau 2:** Caractéristiques des sujets

	Femmes (n = 217)		p	Hommes (n = 235)		p
	PAP (n=117)	Cotonou (n=100)		PAP (n=135)	Cotonou (n=100)	
*Âge (an) (moyenne ± ET)*	38,2±10,1	40,0±9,6	0,188	35,9±10,2	37,8±9,8	0,152
***Niveau socio-économique % (n)***						
*Score du revenu^1^*			0,789			0,908
Faible	39,3 (46)	37,0 (37)		30,4 (41)	29,0 (29)	
Moyen	29,9 (35)	29,0 (29)		34,8 (47)	38,0 (38)	
Élevé	30,8 (36)	34,0 (34)		34,8 (47)	33,0 (33)	
*Niveau d’éducation*			< 0,001			0,018
Aucun ou informel	8,5 (10)	28,0 (28)		3,7 (5)	6,0 (6)	
Primaire	32,5 (38)	35,0 (35)		11,1 (15)	24,0 (24)	
Secondaire et +	59,0 (69)	37,0 (37)		85,2 (115)	70,0 (70)	
***Alimentation et mode de vie***						
*Schémas alimentaires^2^%(n)*			0,263			0,084
Traditionnel	69,6 (78)	76,5 (75)		60,6 (80)	71,9 (64)	
Transitionnel	30,4 (34)	23,5 (23)		39,4 (52)	28,1 (25)	
*Score de consommation d’alcool (moyenne ± ET)*	0,4 ± 0,9	1,5 ± 1,5	< 0,001	1,6 ± 1,4	1,9 ± 1,3	0,087
*Consommation de boissons alcoolisées%(n)*			< 0,001			0,124
Nulle	80,2 (89)	47,0 (47)		38,2 (47)	30,0 (30)	
Faible à modérée/sporadique à dose non excessive	16,2 (18)	16,0 (16)		33,3 (41)	28,0 (28)	
Faible à modérée maissporadiquement élevée	3,6 (4)	29,0 (29)		21,1 (26)	35,0 (35)	
Élevée	0	8,0 (8)		7,3 (9)	7,0 (7)	
*Tabagisme %(n)*			0,036			0,753
Non-fumeurs	91,4 (106)	99,0(99)		75,6 (102)	78,0 (78)	
Anciens fumeurs	5,2 (6)	1,0 (1)		17,0 (23)	17,0 (17)	
Fumeurs	3,4 (4)	0		7,4 (10)	5,0 (10)	
***Donnéesbiologiques (moyenne± ET)***						
Tour de taille (cm)	86,4 ± 13,6	91,1 ± 13,1	0,186	79,6 ± 11,6	84,6 ± 12,7	0,003
HOMA-IR	3,8 ± 2,0	2,7 ± 1,8	<0,001	3,3 ± 1,7	2,0 ± 1,5	<0,001
CT/HDL-C	7,4 ± 3,5	4,3 ± 1,1	<0,001	6,1 ± 2,5	4,3 ± 1,2	<0,001
TAS (mmHg)	120,2±22,2	126,9±24,0	0,029	114,0±15,9	121,9±19,0	0,001
TAD (mmHg)	79,6 ± 14,0	75,5 ± 13,8	0,033	75,4 ± 11,3	72,0 ± 11,7	0,027

1 Score défini par analyse en composante principale basé sur les possessions et les services (proxy du revenu) pour l’ensemble des sujets d’une même ville

2 Schémas alimentaires définis par analyse typologique pour l’ensemble des sujets d’une même ville. ET : écart-type; HOMA : Homeostasis Model Assessment; IR : insulino-résistance; CT/HDL-C : ratio du cholestérol total/HDL-cholestérol; TAS : tension artérielle systolique; TAD : tension artérielle diastolique

La fréquence moyenne de consommation des aliments sélectionnés en fonction des modèles alimentaires est présentée au Tableau 1 pour Cotonou et PAP. On y distingue les aliments traditionnels, urbains et occidentaux, dont quelques-uns sont spécifiques et d’autres communs aux deux villes. À partir de l’analyse typologique basée sur la fréquence de consommation de ces aliments au cours de la dernière semaine et après élimination des grappes incluant 10% ou moins des sujets, deux schémas alimentaires contrastés émergent dans chacune des deux villes: un modèle « transitionnel » chez 34,1% et 24% de l’ensemble des sujets respectivement àPAP et à Cotonou et un modèle « traditionnel » chez 62,7% et 69,5% de l’ensemble des sujets respectivement à PAP et à Cotonou. À PAP, les sujets ayant un schéma alimentaire transitionnel rapportent une consommation significativement plus fréquente de presque tous les aliments, qu’ils soient occidentaux, urbains ou même traditionnels, que chez les sujets du schéma traditionnel. À Cotonou, le schéma transitionnel se distingue du schéma traditionnel par une consommation plus fréquente de certains aliments occidentaux (boissons gazeuses, bonbons, frites et hamburgers) et une consommation plus fréquente d’aliments urbains, de même que de certains aliments traditionnels. Néanmoins, la fréquence de consommation de la plupart des aliments occidentaux et urbains est généralement faible, même dans le modèle alimentaire transitionnel, ne dépassant une fois par semaine en moyenne, même dans le modèle alimentaire transitionnel, que dans le cas des boissons gazeuses et de quelques rares aliments. Les sujets ayant un schéma alimentaire transitionnel ont aussi un revenu plus élevédans les deux villes; à Cotonou seulement, les sujets dont le modèle alimentaire est transitionnel ont également un niveau d’éducationplus élevé. À PAP, la proportion de fumeurs est plus élevée parmi les sujets dont l’alimentation est de type transitionnel. Toutefois, aucune différence significative n’est détectéeentre les types alimentaires pour les biomarqueurs de RCM et ce, dans les deux villes (données non présentées).

La prévalence des biomarqueurs en fonction du revenu est présentée dans la Figure 1. Les sujets de PAP ayant un revenu élevé ont significativement plus d’OA et d’insulino-résistance (Figure 1 a). ÀCotonou, l’OA ainsi que l’insulino-résistance tendent à être plus fréquentes parmi les sujets tant de revenu moyen qu’élevé si on compare aux sujets ayant un faible revenu (Figure 1 b). S’agissant du niveau d’éducation, seule la prévalence d’une tension artérielle élevée lui est significativement et inversement associée et ce, seulement à PAP, où elle affecte près de la moitié des sujets sans éducation formelle contre 26,7 % des sujets ayant une éducation primaire et 16,8 % des sujets ayant un niveau d’éducation plus élevé (p = 0,012). À Cotonou, on ne discerne qu’une tendance à une moindre fréquence d’hypertension dans le groupe à plus haut revenu. Lesrésultats des analyses de régression logistique multivariée incluant les biomarqueurs de RCMdont l’OAcomme variables dépendanteset les paramètres du NSE et du mode de vie comme variables indépendantes (âge et sexe comme variables de contrôle) sont résumés au Tableau 3. À PAP, un revenu moyen ou élevé et une consommation élevée d’alcool sont indépendamment et positivement associés à l’OA; un revenu élevé est aussi associé à un risque accru d’insulino-résistance comparativement au faible revenu et le tabagisme augmente le risque d’hypertension. À Cotonou, les sujets les plus éduqués sont à plus haut risque d’insulino-résistance et d’un profil lipidique athérogèneque ceux qui n’ont pas d’instruction mais le revenu n’est pas associé de manière significative aux différents biomarqueurs.

**Tableau 3:** Association des variables socio-économiques et du mode de vie avec les biomarqueurs de RCM; RC(IC, 95%); p

	Obésité abdominale		Insulino-résistance^1^		CT/HDL-C élevé		Tension artérielle élevée	
	Cotonou	PAP	Cotonou	PAP	Cotonou	PAP	Cotonou	PAP
**Revenu**								
faible	1,0	1,0	1,0	1,0	1,0	1,0	1,0	1,0
moyen	2,0(0,9-4,5); 0,105	3,3(1,2-9,1); 0,018	2,6(0,8-8,0); 0,108	0,9(0,4-2,1); 0,882	1,3(0,5-3,1); 0,571	0,7(0,3-1,6); 0,443	1,7(0,7-4,1); 0,267	0,8(0,3-1,9); 0,556
élevé	1,3(0,5-3,3); 0,553	6,9(2,5-19,3); <0,001	1,3(0,4-4,7); 0,708	2,7(1,2-5,9); 0,012	1,3(0,5-3,4); 0,586	1,4(0,6-3,2); 0,450	0,6(0,2-1,8); 0,346	0,9(0,3-2,3); 0,762
**Éducation**								
Aucune	1,0	1,0	1,0	1,0	1,0	1,0	1,0	1,0
Primaire	1,1(0,4-3,0); 0,816	1,0(0,2-5,6); 0,972	4,6(1,0-21,2); 0,049	1,3(0,3-5,2); 0,716	2,5(0,9-6,9); 0,082	0,9(0,2-4,6); 0,937	1,7(0,6-5,2); 0,319	0,6(0,1-3,0); 0,561
≥secondaire	1,9(0,7-5,6); 0,220	2,2(0,4-11,9); 0,363	11,0(2,1-58,3); 0,005	1,1(0,3-4,3); 0,897	4,5(1,3-14,8); 0,014	1,2(0,3-5,5); 0,814	1,7(0,5-6,1); 0,383	1,1(0,2-5,2); 0,929
**Alcool^2^**								
0	1,0	1,0	1,0	1,0	1,0	1,0	1,0	1,0
1	1,2(0,5-2,7); 0,743	1,9(0,8-4,5); 0,172	1,3(0,4-3,7); 0,680	1,4(0,7-3,0); 0,383	1,2(0,5-3,1); 0,650	0,5(0,2-1,1); 0,103	0,7(0,2-1,7); 0,386	1,6(0,6-4,0); 0,336
2	1,7(0,8-3,6); 0,157	0,3(0,1-1,7); 0,170	1,0(0,4-2,6); 0,951	0,8(0,3-2,5); 0,724	1,1(0,5-2,6); 0,745	0,8(0,3-2,2); 0,615	0,7(0,3-1,8); 0,514	0,1(0,01-1,2); 0,072
3	2,6(0,8-8,7); 0,116	6,7(1,1-40,0); 0,036	0,7(0,1-3,9); 0,694	1,0(0,2-7,0); 0,963	1,7(0,5-6,1); 0,394	1,1(0,2-5,6); 0,872	2,0(0,6-7,2); 0,265	0,4(0,03-5,1); 0,483
**Tabac**								
Non-fumeur	1,0	1,0		1,0	1,0	1,0	1,0	1,0
Ancien-fumeur	2,1(0,6-6,5); 0,221	2,1(0,5-9,0); 0,324		1,2(0,4-4,0); 0,782	1,9(0,5-6,5); 0,321	1,2(0,4-3,7); 0,766	3,2(0,9-11,2); 0,070	2,6(0,6-12,55); 0,224
Fumeur	3,3(0,5-23,14); 0,238	2,2(0,5-9,4); 0,303		1,3(0,3-5,3); 0,672	3,6(0,5-26,4); 0,213	0,9(0,2-3,4); 0,825	1,1(0,1-12,8); 0,918	6,3(1,3-31,7); 0,025
**Age**								
<45ans	1,0	1,0	1,0	1,0	1,0	1,0	1,0	1,0
≥45 ans	1,4(0,7-2,7); 0,391	6,0(2,5-14,6); <0,001	3,1(1,3-7,8); 0,014	1,4(0,7-3,0); 0,362	2,8(1,3-6,0); 0,008	1,3(0,5-2,9); 0,594	5,3(2,4-11,9); <0,001	10,9(4,6-25,6); <0,001
**Sexe**								
Homme	1,0	1,0	1,0	1,0	1,0	1,0	1,0	1,0
Femme	1,3(0,6-2,8); 0,475	4,0(1,7-9,6); 0,001	5,9(2,1-16,1); 0,001	1,5(0,7-3,0); 0,263	9,5(3,9-23,3); <0,001	3,9(1,8-8,3); <0,001	1,4(0,6-3,4); 0,452	2,1(0,9-5,1); 0,093

1 Le modèle de régression logistique inclut l’OA, l’âge, la ville, le revenu, le niveau d’éducation, le schéma alimentaire, la consommation d’alcool et le tabagisme (non inclus dans le modèle à Cotonou)

2 0 : Consommation nulle; 1 : consommation faible à modérée/sporadique non excessive; 2 : consommation faible à modérée maissporadiquement élevée; 3 : consommation élevé; RC : Rapport de cotes

Les liens entre l’OA et les autres biomarqueurs de RCM selon deux modèles de régression logistique multivariée sont rapportés au Tableau 4. Un premier modèle n’inclut comme variables indépendantes que l’OA, l’âge et le sexe; le modèle 2 inclut aussi les autres variables d’intérêt. Dans les deux modèles de régression logistique et dans les deux villes, l’OA est associée à un risque significativement augmenté d’insulino-résistance et d’hypertension, ainsi que d’un indice élevé d’athérogénicitémais seulement à Cotonou;les femmes sont à risque accru d’insulino-résistance à Cotonou et d’athérogénicité dans les deux villes par rapport aux hommes. Les rapports de cotespour l’association de l’OA avec les autres biomarqueurs varient peu par rapport à ceux du modèle 1. Comme le montre le Tableau 5, l’ASC de l’OA comme prédicteur des autres biomarqueurs de RCM dans chaque ville, ne varie pas significativement quand les variables du NSE (niveau d’éducation) et du mode de vie (tabagisme) sont ajoutés tout en contrôlant pour l’âge et le sexe.

**Tableau 4:** Association de l’OA avec les biomarqueurs de RCM en contrôlant pour le NSE et le mode de vie à Cotonou et à PAP; RC (IC, 95%); p

	Insulino-résistance^3^		CT/HDL-C élevé		Tension artérielle élevée	
	Cotonou	PAP	Cotonou	PAP	Cotonou	PAP
**Modèle 1^1^**						
**OA**						
(TT≥88cm H; 95 cm F)	6,5(2,8-14,9); <0,001	5,0(2,5-9,9); <0,001	5,8(2,9-11,7); <0,001	2,3(1,0-5,4); 0,057	3,9(1,9-8,0); <0,001	3,2(1,5-6,7); 0,002
**Âge**						
<45 ans	1,0	1,0	1,0	1,0	1,0	1,0
≥45 ans	1,7(0,8-3,9); 0,197	0,8(0,4-1,5); 0,460	1,8(0,9-3,6); 0,077	1,2(0,6-2,7); 0,579	5,0(2,5-10,2); <0,001	7,2(3,4-14,7);<0,001
**Sexe**						
Homme	1,0	1,0	1,0	1,0	1,0	1,0
Femme	3,1(1,3-7,2); 0,008	1,1(0,6-2,0); 0,671	5,5(2,7-10,9); <0,001	3,9(2,0-7,4); <0,001	1,2(0,6-2,5);0,569	1,8(0,9-3,8); 0,091
**Modèle 2^2^**						
**OA** (TT≥88cm H; 95 cm F)	7,6(2,9-19,9); <0,001	5,1(2,3-11,3); <0,001	5,6(2,6-12,2); <0,001	2,4(0,9-6,4); 0,071	4,2(1,9-9,3); <0,001	3,2(1,3-7,8); 0,011
**Éducation**						
Aucune	1,0	1,0	1,0	1,0	1,0	1,0
Primaire	5,5(1,1-28,3); 0,042	1,3(0,3-5,4); 0,726	2,7(0,9-8,0); 0,083	0,9(0,2-4,4); 0,881	1,8(0,6-5,7); 0,299	0,6(0,1-3,0); 0,559
≥ Secondaire	11,2(1,9-65,9); 0,007	0,9(0,2-3,9); 0,920	3,9(1,1-13,9);0,037	1,0(0,2-4,9); 0,953	1,5(0,4-5,4); 0,556	1,0(0,2-5,0); 0,987
**Tabac**						
Non-fumeurs		1,0	1,0	1,0	1,0	1,0
Anciens-fumeurs		1,1(0,3-3,8); 0,877	1,6(0,4-5,9); 0,481	1,1(0,4-3,5); 0,835	2,6(0,7-9,5); 0,162	2,5(0,5-11,5); 0,240
Fumeurs		1,3(0,3-5,3); 0,734	2,5(0,3-22,1);0,408	0,8(0,2-3,0); 0,690	0,8(0,1-9,6); 0,882	5,9(1,1-31,5); 0,039
**Age**						
**<45 ans**	1,0	1,0	1,0	1,0	1,0	1,0
**≥45 ans**	3,4(1,2-9,1); 0,017	0,8(0,4-1,9); 0,652	2,8(1,2-6,3); 0,012	1,0(0,4-2,3); 0,922	5,7(2,4-13,5); <0,001	8,4(3,5-20,6); <0,001
**Sexe**						
Hommes	1,0	1,0	1,0	1,0	1,0	1,0
Femmes	7,4(2,4-22,5); <0,001	1,0(0,5-2,2); 0,933	12,0(4,5-32,2); <0,001	3,4(1,6-7,5); <0,001	1,4(0,6-3,5); 0,496	1,8(0,7-4,5); 0,201

1 Le modèle de régression logistique inclut l’OA, l’âge et la ville

2 Le modèle de régression logistique contient l’OA, l’âge, la ville, le revenu, le niveau d’éducation, le schéma alimentaire, la consommation d’alcool et le tabagisme

3 À Cotonou, le modèle de régression logistique ne contient pas les catégories de tabagisme. RC : rapport de cotes; TT : tour de taille

**Tableau 5:** Aires sous la courbe pour la prédiction des biomarqueurs de RCM; ASC ± erreur-type (IC à 95%)

	Insulino-résistance		CT/HDL-C élevé		Tension artérielle élevée	
	Cotonou (n = 200)	PAP (n = 252)	Cotonou (n = 200)	PAP (n = 252)	Cotonou (n = 200)	PAP (n = 252)
**OA+âge+sexe**	0,771±0,043 (0,686-0,856)^1^	0,658±0,039 (0,581-0,735)	0,764±0,033 (0,699-0,829)	0,705±0,038 (0,631-0,779)	0,757±0,039 (0,680-0,833)	0,820±0,032 (0,758-0,883)
**OA + âge + sexe + niveau d’éducation + tabagisme**	0,800±0,041 (0,719-0,882)	0,660±0,040 (0,582-0,737)	0,785±0,032 (0,721-0,848)	0,687±0,038 (0,613-0,760)	0,778±0,036 (0,707-0,849)	0,825±0,030 (0,766-0,849)
**p^2^**	0,626	0,971	0,648	0,738	0,692	0,909

1 Toutes les ASC sont significatives à p < 0,01

2 p de la comparaison des ASC entre (OA+âge+sexe) et (OA+âge+sexe+niveau d’éducation+tabagisme); significatif à p < 0,05; OA : obésité abdominale

## Discussion

La présente étude dans deux groupes de sujets apparemment sains et apparentés génétiquement mais vivant dans deux environnements différents, Cotonou et PAP, visait àcerner la relation entre le mode de vie et les biomarqueurs de RCM, ainsi qu’entre l’OA et les autres biomarqueurs. Le NSE et le mode de vie, excepté l’alimentation, sonteffectivement associés au RCM dans chaque ville, bien quede manière différente. Toutefois, le type alimentaire traditionnel ou « transitionnel » (vers une alimentation occidentalisée) n’est pas associé de manière significative au RCM. L’OA telle que définie par des valeurs-seuils spécifiques de TT, est fortement associée aux autres biomarqueurs et cette association n’est pas modifiée par le NSE ou le mode de vie. Les sujets ayant un revenu élevé sont à plus haut RCM mais seulement à PAP, tandis qu’à Cotonou c’est le niveau d’instruction qui s’associe positivement à ce risque. Ceci suggère une différence dans l’environnement social et économique entre les deux villes [27]. Dans la littérature, le NSE, incluant le revenu et l’éducation, a été surtout étudié dans son association avec l’obésité (générale et abdominale). Cette relationvarie en fonction notamment du niveau de développement économique du pays. Dans les pays industrialisés, un NSE élevé est négativement associé à l’obésité, alors que cette relation s’inverseprogressivement dans les pays à faible ou moyen revenu pour basculer vers les strates de faible NSE [28, 29]. À cause de l’accélération de la transition nutritionnelle et de la mondialisation, on note maintenant que dans les pays en développement, l’obésité et le RCM se retrouventaussi bien chez les sujets à revenu faible qu’élevé [1, 30, 31]. L’association de l’éducation avec les biomarqueurs de RCM dans les pays en développement n’est pas stable; elle est positive dans certaines populations et négative dans d’autres où l’éducation apparaît plutôt comme facteur protecteur contre l’obésité et le RCM [27, 32, 33]. Dans ce sens, la fréquence supérieure de sujets ayant un niveau d’instruction élevé à PAP comparativement à Cotonou pourrait exercer un effet protecteur contre l’effet obésogène du revenu, ce qui pourrait expliquerune prévalence plus faible d’OA à PAP qu’à Cotonou. Dans leur étude chez 143 258 femmes en âge de procréer dans neuf pays d’Amérique latine et des Caraïbes, Joen et al. [34] notaientque la relation entrel’éducation et lesurpoids/obésité était non-linéaire et qu’elle était modulée par le stadede la transition nutritionnelle des pays. En effet, ils ont observé que le risque de surpoids augmentait avec les années d’études pour atteindre un point où cette tendance s’inversaitet que ce point d’inflexion étaitplus rapidement atteint dansles pays où la transition nutritionnelle était plus poussée. Nos données portent à penser que PAP en est à un stade plus avancé de cette transition nutritionnelle, comme en témoigne également la proportion de personnes ayant une alimentation de type « transitionnel » qui tend à être plus élevée à PAP qu’à Cotonou.

Le tabagisme estpeu fréquent chez les sujets de la présente étude mais il estnéanmoins associé au risque d’hypertension, au moins à PAP. Un faible taux de tabagisme était également rapporté dans l’enquête démographique et de santé menée au Bénin 2011 [35]. En revanche, à PAP, une enquête dans l’aire métropolitaine réalisée en 2003 rapportait une prévalence du tabagisme beaucoup plus élevée que dans notre étude [21]; il pourrait y avoir eu une diminution du tabagisme dans l’intervalle. Le risque d’hypertension associé au tabagisme est rapporté dans de nombreuses études, dont INTERHEART, réalisée dans 52 pays, qui démontrait que le tabagisme multipliait par 2,5 le risque d’hypertension et trois fois le risque d’infarctus du myocarde [36]. Quant à l’alcool, une consommation élevée est significativement associée à l’OA chez les Haïtiens mais non chez les Béninois. Un des points forts de notre étude est d’avoir considéré la consommation d’alcool d’après un score composite incluant la consommation totale moyenne d’alcool et la quantité maximale ingérée en une seule occasion, comme le suggère l’OMS. D’après la littérature, une consommation faible à modérée, excluant une consommation sporadique élevée, aurait un effet bénéfique sur le RCM [37, 38]. L’association d’une consommation élevée d’alcool avec l’OA s’expliquerait essentiellement par l’apport élevé en énergie provenant de l’alcool [39]. Outre la contribution des facteurs socio-économiques, on s’attendait à ce que les habitudes alimentaires, élément important du mode de vie, soient en lien avec le RCM, comme l’ont démontré de nombreuses études [40, 41]. Plus précisément, nous postulions qu’une alimentation plus occidentalisée (le schéma alimentaire « transitionnel ») serait associéeà un profil de RCM plus défavorable. Toutefois, en contrôlant pour l’âge, le sexe et le NSE (proxy du revenu et niveau d’instruction), il n’y a pas d’association significative du modèle alimentaire avec les biomarqueurs de RCM, même si dansles deux villes, le schéma alimentaire transitionnel estplus fréquent parmi les sujets de NSE élevé. Les modèles alimentaires ont été obtenus à partir de l’analyse d’un questionnaire de fréquence de consommation d’aliments typiquement occidentaux, urbains ou traditionnels, mais sans tenir compte des quantités consommées, ce qui constitue une limite à notre étude. Des rappelsalimentaires quantitatifs, complets et répétésauraient peut-être permis de mettre en évidence des associations significatives avec les biomarqueurs de risque. Par ailleurs, la nomenclature des deux schémas alimentaires conserve une part d’arbitraire dans le choix des solutions de l’analyse typologique, bien que notre équipe ait démontré que le régime alimentaire traditionnel est plus adéquat en termes de micronutriments et plus conforme aux directives de l’OMS pour la prévention des maladies chroniques chez les Haïtiens vivant à Montréal (Canada) [42] de même que chez les Béninois issus de différentes localités [43, 44], mais ceci, sur la base de rappels alimentaires quantitatifs répétés.

Malgré la relation entre le NSE ou le mode de vie etla prévalence de l’OA (du moins à PAP) et des autres biomarqueurs de RCM, ces facteurs n’amplifient ni ne réduisent le degréd’association de l’OA avec les autres biomarqueurs. Ceci se confirme dans l’analyse comparative des ASC qui montre quel’OA est prédictive du RCM et que les facteurs environnementaux n’améliorent pas, pour autant, ce pouvoir prédictif. La relation entre l’OA et le RCM, d’insulino-résistance, duprofil lipidique athérogène, de l’hypertension ou de l’inflammation, ou du syndrome métabolique, n’est plus à démontrer [4, 45–47]. L’originalité, la force de notre étude a été d’examiner l’association de l’OA avec les biomarqueurs de RCM en utilisant des valeurs-seuils de TT spécifiques pour la population étudiée (88 cm chez les hommes et 95 cm chez les femmes) et en déterminant ces valeurs-seuils d’après leur valeur prédictive des biomarqueurs les plus fortement prévalents dans ces populations, à savoir, l’insulino-résistance, l’hypertension et un profil lipidique athérogène d’après l’indice d’athérogénicité [12]. Si on utilise les seuils génériques de TT pour définir l’OA (= 80cm chez les femmes et = 94 cm chez les hommes) [16] plutôt que les seuils spécifiques, le risque d’athérogénicité à PAP [RC (95% IC) = 8,(33-20,2); p < 0001] et d’hypertension à Cotonou [RC (95% IC) = 11,0 (36-33,9); p < 0,001] augmente (données non présentées). Dans d’autres études chez des populations noires, les seuils génériques de TT paraissaient également peu adéquats pour prédire le RCM et ce, particulièrement chez les femmes [9, 10, 48]. Ceci suggère de mesurer le TT en utilisant des valeurs-seuils spécifiques afin de mieux prédire le RCM chez les populations noires. Dans ce même sens, Agueh et al. [7] dans une étude longitudinale incluant les sujets de Cotonou de notre étude, outre des groupes d’autres sites du Bénin, rapportaient que les valeurs-seuils de TT (81cm chez les hommes et 90 cm chez les femmes) pour prédire au moins une composante du syndrome métabolique restaient stables après un suivi de quatre ans. Ces seuils sont plus faibles par rapport à ceux que nous avons déterminés, mais les biomarqueurs de RCM n’étaient pas les mêmes puisque cette autre étude considérait le syndrome métabolique dans son ensemble plutôt que les biomarqueurs les plus fréquents [10]. Néanmoins, dans cette étude comme dans d’autres chez des Noirs, les valeurs-seuils sont inversées par rapport à celles établies pour des populations d’origine européenne, ces valeurs étant plus élevées chez les femmes que chez les hommes. Certaines limites de la présente étudedoivent être mentionnées, dont son caractère transversal qui ne permet pas d’établir de liens de causalité entre les variables de NSE et de mode de vie et les biomarqueurs de RCM;seules des associations significatives peuvent être démontrées. En outre, nous n’avons considéré, ni l’activité physique ni la sédentarité commefacteurs de la transition nutritionnelle pouvant contribuer au RCMet éventuellement à la divergence entre Cotonou et PAP dans la prévalence des biomarqueurs de RCM [49]. Bien que nous ayons identifié le rôle de l’OA comme prédicteur du RCM, des études longitudinales et sur de plus grands échantillonssont nécessaires pour vérifier le lien de causalité et pour valider les valeurs-seuils spécifiques définies par la présente étude.

## Conclusion

En dehors des facteurs génétiques susceptibles de prédisposer les sujets de ces deux villes au même RCM, le mode de vie et le NSE affectent le niveau de RCM, mais ils ne modulent pas la relation de l'OA et les autres biomarqueurs de risque. Ainsi les seuils de TT définis pour ces population trouvent ici confirmation, bien que leur validation nécessite davantage d'études, surtout longitudinales.

### Etat des connaissances actuelle sur le sujet

- Le risque cardiométabolique continue d’augmenter dans les pays à faible et à moyen revenu, en partie à cause de la transition nutritionnelle;
- L’obésité abdominale est associée aux aberrations métaboliques, mais les études ont surtout porté sur des Caucasiens et des Afro-Américains.

### Contribution de notre étude à la connaissance

- L’obésité abdominale, définie d’après des seuils spécifiques de tour de taille (88cm chez les hommes et 94 cm chez les femmes), est positivement associée au biomarqueurs de risque les plus fréquents (résistance à l’insuline, ratio CT/HDL-cholestérol élevé et hypertension artérielle) dans deux populations génétiquement apparentées : les Béninois (Cotonou) et les Haïtiens (Port-au-Prince);
- Un niveau socio-économique élevé et la consommation d’alcool et de tabac aggravent les biomarqueurs de risque cardiométabolique, mais ils n’influencent pas la relation entre l’obésité abdominale et les autres biomarqueurs de risque cardiométabolique dans ces deux groupes de population;
- Deux schémas alimentaires contrastés ont pu être identifiés dans ces deux villes, l’un traditionnel et l’autre transitionnel, ce dernier comportant davantage d’aliments « occidentaux ».

## References

[1] Swinburn BA, Sacks G, Hall KD, McPherson K, Finegood DT, Moodie ML (2011). The global obesity pandemic: shaped by global drivers and local environments. Lancet.

[2] Popkin BM, Adair LS, Ng SW (2012). Global nutrition transition and the pandemic of obesity in developing countries. Nutr Rev..

[3] Yusuf S, Reddy S, Ounpuu S, Anand S (2001). Global burden of cardiovascular diseases: part I: general considerations, the epidemiologic transition, risk factors, and impact of urbanization. Circulation.

[4] Despres JP (2012). Body fat distribution and risk of cardiovascular disease: an update. Circulation.

[5] Dalal S, Beunza JJ, Volmink J, Adebamowo C, Bajunirwe F, Njelekela M (2011). Non-communicable diseases in sub-Saharan Africa: what we know now. International journal of epidemiology.

[6] Sossa C, Delisle H, Agueh V, Makoutode M, Fayomi B (2012). Four-Year Trends in Cardiometabolic Risk Factors according to Baseline Abdominal Obesity Status in West-African Adults: The Benin Study. Journal of obesity.

[7] Agueh V, Sossa C, Ouendo D, Paraizo N, Azandjemè C, Kpozehouen A (2015). Determination of the optimal waist circumference cut-off points in Benin adults. Open Journal of Epidemiology.

[8] Motala AA, Esterhuizen T, Pirie FJ, Omar MA (2011). The prevalence of metabolic syndrome and determination of the optimal waist circumference cutoff points in a rural South african community. Diabetes care.

[9] Hoebel S, Malan L, Botha J, Swanepoel M (2014). Optimizing waist circumference cut-points for the metabolic syndrome in a South African cohort at 3-year follow-up: the SABPA prospective cohort. Endocrine.

[10] El Mabchour A, Delisle H, Vilgrain C, Larco P, Sodjinou R, Batal M (2015). Specific cut-off points for waist circumference and waist-to-height ratio as predictors of cardiometabolic risk in Black subjects: a cross-sectional study in Benin and Haiti. Diabetes, metabolic syndrome and obesity : targets and therapy.

[11] Sodjinou R, Agueh V, Fayomi B, Delisle H (2008). Obesity and cardio-metabolic risk factors in urban adults of Benin: relationship with socio-economic status, urbanisation, and lifestyle patterns. BMC public health.

[12] EL Mabchour A, Delisle H, Vilgrain C, Larco P, Sodjinou R, Batal M (2015). Abdominal obesity and other cardiometabolic risk biomarkers in men and in women in two urban population groups undergoing the nutrition transition: Cotonou (Benin) and Port-Au-Prince (Haiti). Integr Obes Diabetes.

[13] Lohman TG, Roche AF, Martorell R (1988). Anthropometric standardization reference manual.

[14] National Cholesterol Education Program Expert Panel on Detection E, Treatment of High Blood Cholesterol in A (2002). Third Report of the National Cholesterol Education Program (NCEP) Expert Panel on Detection, Evaluation, and Treatment of High Blood Cholesterol in Adults (Adult Treatment Panel III) final report. Circulation.

[15] Matthews DR, Hosker JP, Rudenski AS, Naylor BA, Treacher DF, Turner RC (1985). Homeostasis model assessment: insulin resistance and beta-cell function from fasting plasma glucose and insulin concentrations in man. Diabetologia..

[16] Alberti KG, Eckel RH, Grundy SM, Zimmet PZ, Cleeman JI, Donato KA (2009). Harmonizing the metabolic syndrome: a joint interim statement of the International Diabetes Federation Task Force on Epidemiology and Prevention; National Heart, Lung, and Blood Institute; American Heart Association; World Heart Federation; International Atherosclerosis Society; and International Association for the Study of Obesity. Circulation.

[17] Organisation Mondiale de la Santé (2002). Questionnaire STEPS Pour les facteurs de risque des MNT (Version de base et élargie, V 1.4).

[18] Vyas S, Kumaranayake L (2006). Constructing socio-economic status indices: how to use principal components analysis. Health policy and planning.

[19] Houweling TA, Kunst AE, Mackenbach JP (2003). Measuring health inequality among children in developing countries: does the choice of the indicator of economic status matter?. International journal for equity in health.

[20] Institut National de la Statistique et de l’Analyse Économique (INSAE) et ORC Macro (2002). Enquête démographique et de santé au Bénin 2001.

[21] Institut Haitien des Statistiques et d'Informatique (2003). Enquête sur les conditions de vie en Haïti -2001.

[22] Newby PK, Muller D, Tucker KL (2004). Associations of empirically derived eating patterns with plasma lipid biomarkers: a comparison of factor and cluster analysis methods. The American journal of clinical nutrition.

[23] Babor TF, Higgins-Biddle JC, Saunders JB, Monteiro MG (2001). The alcohol use disorders identification test (AUDIT) instrument, Guidelines for use in primary care.

[24] Gmel G, Kuntsche E, Rehm J (2011). Risky single-occasion drinking: bingeing is not bingeing. Addiction.

[25] Greenfield TK, Nayak MB, Bond J, Ye Y, Midanik LT (2006). Maximum quantity consumed and alcohol-related problems: assessing the most alcohol drunk with two measures. Alcoholism, clinical and experimental research.

[26] Zweig MH, Campbell G (1993). Receiver-operating characteristic (ROC) plots: a fundamental evaluation tool in clinical medicine. Clinical chemistry.

[27] Monteiro CA, Conde WL, Popkin BM (2001). Independent effects of income and education on the risk of obesity in the Brazilian adult population. The Journal of nutrition.

[28] Sobal J, Stunkard AJ (1989). Socioeconomic status and obesity: a review of the literature. Psychological bulletin.

[29] Zeba AN, Delisle HF, Renier G, Savadogo B, Baya B (2012). The double burden of malnutrition and cardiometabolic risk widens the gender and socio-economic health gap: a study among adults in Burkina Faso (West Africa). Public health nutrition.

[30] Hawkes C (2006). Uneven dietary development: linking the policies and processes of globalization with the nutrition transition, obesity and diet-related chronic diseases. Globalization and health.

[31] Dinsa GD, Goryakin Y, Fumagalli E, Suhrcke M (2012). Obesity and socioeconomic status in developing countries: a systematic review. Obesity reviews : an official journal of the International Association for the Study of Obesity.

[32] Aitsi-Selmi A, Bell R, Shipley MJ, Marmot MG (2014). Education modifies the association of wealth with obesity in women in middle-income but not low-income countries: an interaction study using seven national datasets, 2005-2010. PloS one.

[33] Gyakobo M, Amoah AG, Martey-Marbell DA, Snow RC (2012). Prevalence of the metabolic syndrome in a rural population in Ghana. BMC endocrine disorders.

[34] Haram J, Salinas D, Baker DP (2015). Non-linear education gradient across the nutrition transition: mothers' overweight and the population education transition. Public health nutrition.

[35] Institut National de la Statistique et de l’Analyse Économique (INSAE) et ICF International (2013). Enquête Démographique et de Santé 2011-2012.

[36] Teo KK, Ounpuu S, Hawken S, Pandey MR, Valentin V, Hunt D (2006). Tobacco use and risk of myocardial infarction in 52 countries in the INTERHEART study: a case-control study. Lancet.

[37] Rehm J, Baliunas D, Borges GL, Graham K, Irving H, Kehoe T (2010). The relation between different dimensions of alcohol consumption and burden of disease: an overview. Addiction.

[38] Rehm J, Patra J (2012). Different guidelines for different countries? On the scientific basis of low-risk drinking guidelines and their implications. Drug and alcohol review.

[39] Schroder H, Morales-Molina JA, Bermejo S, Barral D, Mandoli ES, Grau M (2007). Relationship of abdominal obesity with alcohol consumption at population scale. European journal of nutrition.

[40] Liu J, Hickson DA, Musani SK, Talegawkar SA, Carithers TC, Tucker KL (2013). Dietary patterns, abdominal visceral adipose tissue, and cardiometabolic risk factors in African Americans: the Jackson heart study. Obesity.

[41] Dhingra R, Sullivan L, Jacques PF, Wang TJ, Fox CS, Meigs JB (2007). Soft drink consumption and risk of developing cardiometabolic risk factors and the metabolic syndrome in middle-aged adults in the community. Circulation.

[42] Desilets MC, Rivard M, Shatenstein B, Delisle H (2007). Dietary transition stages based on eating patterns and diet quality among Haitians of Montreal, Canada. Public health nutrition.

[43] Delisle H, Ntandou-Bouzitou G, Agueh V, Sodjinou R, Fayomi B (2012). Urbanisation, nutrition transition and cardiometabolic risk: the Benin study. The British journal of nutrition.

[44] Delisle H, Agueh V-D, Sodjinou R, Ntandou-Bouzitou G, Daboné C, Preedy VR, Hunter L-A, Patel VB (2013). Dietary Quality and the Nutrition Transition in Sub-Saharan Africa. Diet Quality, Nutrition and Health.

[45] Gaillard T (2014). Consequences of Abdominal Adiposity within the Metabolic Syndrome Paradigm in Black People of African Ancestry. Journal of clinical medicine.

[46] Doumatey AP, Lashley KS, Huang H, Zhou J, Chen G, Amoah A (2010). Relationships among obesity, inflammation, and insulin resistance in African Americans and West Africans. Obesity.

[47] Ntandou G, Delisle H, Agueh V, Fayomi B (2009). Abdominal obesity explains the positive rural-urban gradient in the prevalence of the metabolic syndrome in Benin, West Africa. Nutrition research.

[48] Katzmarzyk PT, Bray GA, Greenway FL, Johnson WD, Newton RL, Ravussin E (2011). Ethnic-specific BMI and waist circumference thresholds. Obesity.

[49] Sossa C, Delisle H, Agueh V, Sodjinou R, Ntandou G, Makoutode M (2013). Lifestyle and dietary factors associated with the evolution of cardiometabolic risk over four years in West-African adults: the Benin study. Journal of obesity.

